# Pediatric obstructive sleep apnea: knowledge, attitude, and practice among pediatric dentists in Egypt: a cross-sectional study

**DOI:** 10.1186/s12903-025-06944-w

**Published:** 2025-09-23

**Authors:** Mariam Mohsen Aly, Marwa Aly Elchaghaby, Yasmin Mohamed Yousry

**Affiliations:** Pediatric Dentistry and Dental Public Health, Faculty of Dentistry, Cairo University, Giza, Egypt

**Keywords:** Attitude, Knowledge, Obstructive sleep apnea, Pediatric dentists, Practice

## Abstract

**Background:**

Pediatric dentists can play a crucial role in detecting, referring, counselling, and treating patients with obstructive sleep apnea (OSA). Understanding their knowledge and attitudes towards OSA is crucial for effective healthcare delivery.

**Objective:**

This study aimed to assess the knowledge, attitude, and practice of pediatric dentists in Egypt toward obstructive sleep apnea in children.

**Methods:**

This cross-sectional study used a web-based structured questionnaire generated via Google Forms based on a previously validated questionnaire. The link for the questionnaire was circulated to participants through emails and professional group forums. The questionnaire comprised an introductory paragraph and four sections: demographic, knowledge, attitude, and practice for data collection.

**Results:**

A total of 362 pediatric dentists participated in this online survey. Adequate knowledge was detected in 310 (85.64%) pediatric dentists, and 343 (94.75%) demonstrated a positive attitude toward OSA. In contrast, inadequate practices were observed in 274 (75.69%) pediatric dentists. Concerning the correlation between the knowledge, attitude, and practice and demographic data, a weak negative correlation with statistical significance (*p*-value = 0.047186) was detected between practice and age. A weak positive correlation was also detected between knowledge and years of experience, with a statistical significance (*p*-value = 0.011848).

**Conclusions:**

Pediatric dentists in Egypt seem to have adequate knowledge and a positive attitude toward different domains of pediatric OSA, but their practice was inadequate. Participants’ knowledge showed a weak favourable correlation with years of experience; meanwhile, their practice has an adverse weak relationship with age.

**Trial registration:**

The current study was registered on May 6, 2025, with the identifier number NCT06970873 on clinicaltrials.gov.

**Supplementary Information:**

The online version contains supplementary material available at 10.1186/s12903-025-06944-w.

## Introduction

Despite being one of the most common sleep disorders, Obstructive sleep apnea (OSA) is frequently underdiagnosed and untreated. Based on the American Thoracic Society, OSA is defined as a breathing disorder during sleep, featured by prolonged partial upper airway obstruction and/or interrupted complete obstruction, disrupting normal ventilation during sleep and usual sleep patterns [1].

The global prevalence of OSA in adults ranges from 9% to 38%, depending on the diagnostic criteria applied. Moreover, a recent systematic review reported that the prevalence of pediatric OSA based on studies published between 2016 and 2023 ranges from 12.8% to 20.4% [2, 3]. The prevalence of OSA among children in Egypt is not definitively established, but studies suggest it is a significant health concern, especially in certain populations, such as children with bronchial asthma or Down syndrome [4, 5].

Pediatric OSA varies from adult OSA since it displays features specific to growing persons. It is characterized by the partial or total obstruction of the airways, leading to a stoppage or continued airflow restriction during sleep. Compromised ventilation may accompany oxyhemoglobin desaturation, hypercapnia, and changed sleep patterns [6, 7].

Pediatric OSA can cause various clinical manifestations, including daytime and nighttime symptoms. There are multiple signs and symptoms during nighttime, such as snoring, stops in breathing, restless sleep, inconsistent chest movement, uncommon sleeping positions, repeated awakenings, and enuresis. Morning signs and symptoms include nasal obstruction, mouth breathing, neurocognitive troubles, behavior problems, inadequate school performance, daytime sleepiness, and poor growth [8].

Untreated OSA can cause significant health concerns, such as hypertension, cardiovascular diseases, diabetes mellitus, stroke, a reduction in quality of life, and even death. Knowledge of this point is fundamental for healthcare workers, as most OSA complications can be prevented if detected and managed appropriately at the proper time [9].

The complexity of OSA requires a multidisciplinary approach for effective screening and management, placing dental professionals in a crucial role. Dentists frequently encounter early signs of OSA, such as airway obstruction and bruxism. Furthermore, dentists can be the earliest line of OSA diagnosis by assessing the size of the uvula, the size and position of the tongue, and the length of the soft palate during regular clinical examinations, which may be unnoticed in a general medical examination [9, 10].

Dentists must provide timely, consistent care while working closely with physicians by executing a suitable screening protocol for OSA, tailed by an appropriate referral to sleep physicians for sleep review and treatment. Considering OSA’s prevalence and adverse effects on health, healthcare workers have become more keen on screening and managing OSA in several countries [11–13].

Still, former studies have shown that knowledge and practice of OSA among dentists were unsatisfactory, which eventually increases the prevalence of undiagnosed cases of OSA [7, 9, 13, 14].

To the best of the authors’ knowledge, no studies have been conducted in Egypt to evaluate pediatric dentists’ knowledge, attitude, and practice toward pediatric obstructive sleep apnea. Assessing their understanding and practice can encourage their confident participation in the interdisciplinary management of pediatric OSA and aid in identifying areas in need of continuing education. So, the present study aimed to assess pediatric dentists’ knowledge, attitudes, and practices toward pediatric obstructive sleep apnea in Egypt.

## Subjects and methods

### Study design and objectives

This observational cross-sectional study was performed to assess pediatric dentists’ knowledge, attitudes, and practices toward pediatric obstructive sleep apnea in Egypt.

### Target population

#### Inclusion criteria

Licensed specialized pediatric dentists, graduated from Egyptian universities.

#### Exclusion criteria

General dentists not specialized in pediatric dentistry and dentists who declined to consent to the survey.

### Ethical concerns

Before starting the study, an ethical agreement was granted by the Research Ethics Committee, Faculty of Dentistry, Cairo University, Egypt, with an approval number (22-2-25). The study was conducted in accordance with the Declaration of Helsinki.

Moreover, the present study followed STROBE guidelines for reporting observational studies and was registered on clinicaltrials.gov with the identifier NCT06970873.

### Sample size estimate

Based on the findings of Nair et al. [15], in which the knowledge of pediatric dentists regarding OSA among children was (80.3%), the expected sample size (*n*) was a total of (362) cases by implementing a (95%) confidence interval. The Epi Info application was employed to calculate the sample size.

### Questionnaire structure and data collection procedure

The questionnaire used in the present study is a pre-prepared, validated English questionnaire from a previous study on pediatric dentists by Nair et al. [15]. This web-based, self-administered, structured questionnaire was generated via the Google Forms platform, and the link for the questionnaire was sent to the study participants through email and professional group forums. The questionnaire comprised four sections (demographic, knowledge, attitude, and practice) with a series of multiple-choice questions along with an introductory paragraph.

The study’s goal was explained in depth in the introduction, emphasizing that participation would be anonymous and voluntary to guarantee the confidentiality and privacy of the information gathered. A consent agreement was obtained electronically before accessing the survey by checking the box to confirm the agreement.

The demographic section includes five questions to collect information regarding participant age, gender, nationality, years of practice, and practice setting. To gauge pediatric dentists’ level of OSA knowledge, the knowledge section included seventeen questions regarding the definition of OSA, the prevalence with age and gender predilection, the commonly affected age group, the general findings, the clinical features, risk factors, the diagnosis using polysomnography, as well as the treatment and referral of OSA cases.

Seven questions in the attitude section focused on the role of pediatric dentists in identifying and managing OSA in children, screening for OSA based on snoring history, the necessity of including information about OSA in undergraduate curricula, and making OSA screening an essential part of clinical examination.

The five questions in the practice section assessed whether pediatric dentists had previously made oral appliances to treat OSA, strategies in history taking, including inquiries to exclude OSA, and whether they had ever attended OSA-related continuing education courses. Two weeks after the questionnaire link distribution, a reminder was sent to encourage participation and to thank those who had already participated. After completing data collection, responses were exported to an Excel sheet and analyzed.

### Scoring of questionnaire responses

The questionnaire’s scoring was based on a previous study conducted by Nair et al. [15], which served as the foundation for the assessment procedure and score categorization.

Regarding the knowledge questions, there were three possible answers: “true,” “false,” and “do not know.” One point was given for every correct response, and no points were given for incorrect answers. The “do not know” option was added to reduce guessing and was graded as an incorrect response.

The attitude’s five-point scale was converted into a dichotomized approach following previous studies by Alzahrani et al. [13]; Kale et al. [14]; and Nair et al. [15] to simplify the data interpretation and to establish a clear threshold for practical use. “Strongly agree” and “agree”, which represent a positive attitude, were granted one point. While “neutral”, “disagree”, and “strongly disagree”, which represent a negative attitude, were awarded no points.

The response possibilities were yes or no in the practice section, where a response of “yes” was awarded one point, indicating a good practice. In contrast, no point was awarded for “no” response, indicating a poor practice.

The overall percentage of dentists who correctly answered each question was combined to interpret the results properly according to Nair et al. [15] and Chauhan et al. [16], as follows:

- Adequate knowledge is considered when at least 50% of pediatric dentists provide accurate answers, while inadequate knowledge is considered when less than half of pediatric dentists respond incorrectly.
- A positive attitude is achieved when 50% or more of participants respond with agree or strongly agree. In contrast, a negative attitude is achieved when less than half of the participants respond with “neutral,” “disagree,” or “strongly disagree.”
- Good practice is manifested when at least 50% of dentists treat OSA-related disorders. In contrast, poor practice is manifested when less than half of the dentists don’t treat OSA-related disorders.
- Finally, the overall percentage of correct answers was calculated independently for the knowledge, attitude, and practice sections. The overall knowledge of the participants was considered adequate, their attitude was considered positive, and their practice was considered good when more than 50% of the questions were answered correctly.

### Statistical analysis

The statistical analysis program Statistical Package for Social Sciences (SPSS) version 22.0 was employed to perform all the statistical analyses. Descriptive statistics were used to calculate the numbers, frequencies, and percentages for each category of categorical data. The Phi correlation coefficient was used to evaluate the correlation between knowledge, attitude, practice, and gender, ranging from − 1 to 1, with a statistical significance set at *p* ≤ 0.05. The correlations between knowledge, attitude, practice, and age, as well as years of experience, were evaluated using the rank biserial correlation, ranging from − 1 to 1, with a statistical significance set at *p* ≤ 0.05. Finally, Goodman and Kruskal’s Lambda was utilized to assess the correlation between knowledge, attitude, practice, and practice sector as well as nationality, ranging from 0 to 1, with a statistical significance set at *p* ≤ 0.05.

## Results

### Demographics

Of the 362 pediatric dentists who participated in this online survey, 207(57.18%) were Females, and 155 (42.82%) were Males. Most of the respondents, 234 (64.64%), were in the age range 25–34 years, and only 6 (1.66%) were more than 54 years old. Among the respondents, the largest group, 328 (90.61%), consisted of Egyptian dentists. Regarding the practice sector, about 175 (48.34%) respondents were engaged in academic work. In comparison, private work was chosen by 111 (30.66%), and primary health care hospitals by 66 (18.23%). Concerning the experience years of the participants, the majority of the participants, 141 (38.95%), had been practicing for 5 to 10 years, followed by 98 (27.07%) possessing experience of 10 to 20 years, and 91 (25.14%) less than five years, as shown in Table 1.

**Table 1:** The participants’ demographic characteristics

Background characteristics	Variable	Number	Percentages (%)
Gender	Male	155	42.82
	Female	207	57.18
Age	25–34 Y	234	64.64
	35–44 Y	110	30.39
	45–54 Y	12	3.31
	More than 54 Y	6	1.66
Nationality	Egyptian	328	90.61
	Palestinian	6	1.66
	Saudi	5	1.38
	Sudanese	13	3.59
	Syrian	6	1.66
	Yemeni	4	1.10
Practice sector	Academic work	175	48.34
	Primary healthcare hospital	66	18.23
	Private	111	30.66
	Others	10	2.76
Years of experience	Less than 5 years	91	25.14
	From 5 to 10 years	141	38.95
	From 10 to 20 years	98	27.07
	More than 20 years	32	8.84

### Responses to the knowledge section

The majority of the participants provided good knowledge concerning the correct definition of OSA (Q. No. 1), age predilection (Q. No.3), symptom of OSA (Q. No.6), the clinical features of OSA (Q. No. 8, and 10), risk factors for OSA (Q. No. 7 and 11), as well as the treatment of OSA with oral appliance (Q. No. 13)., and referral of OSA cases (Q. No. 14).

**Fig. 1:**
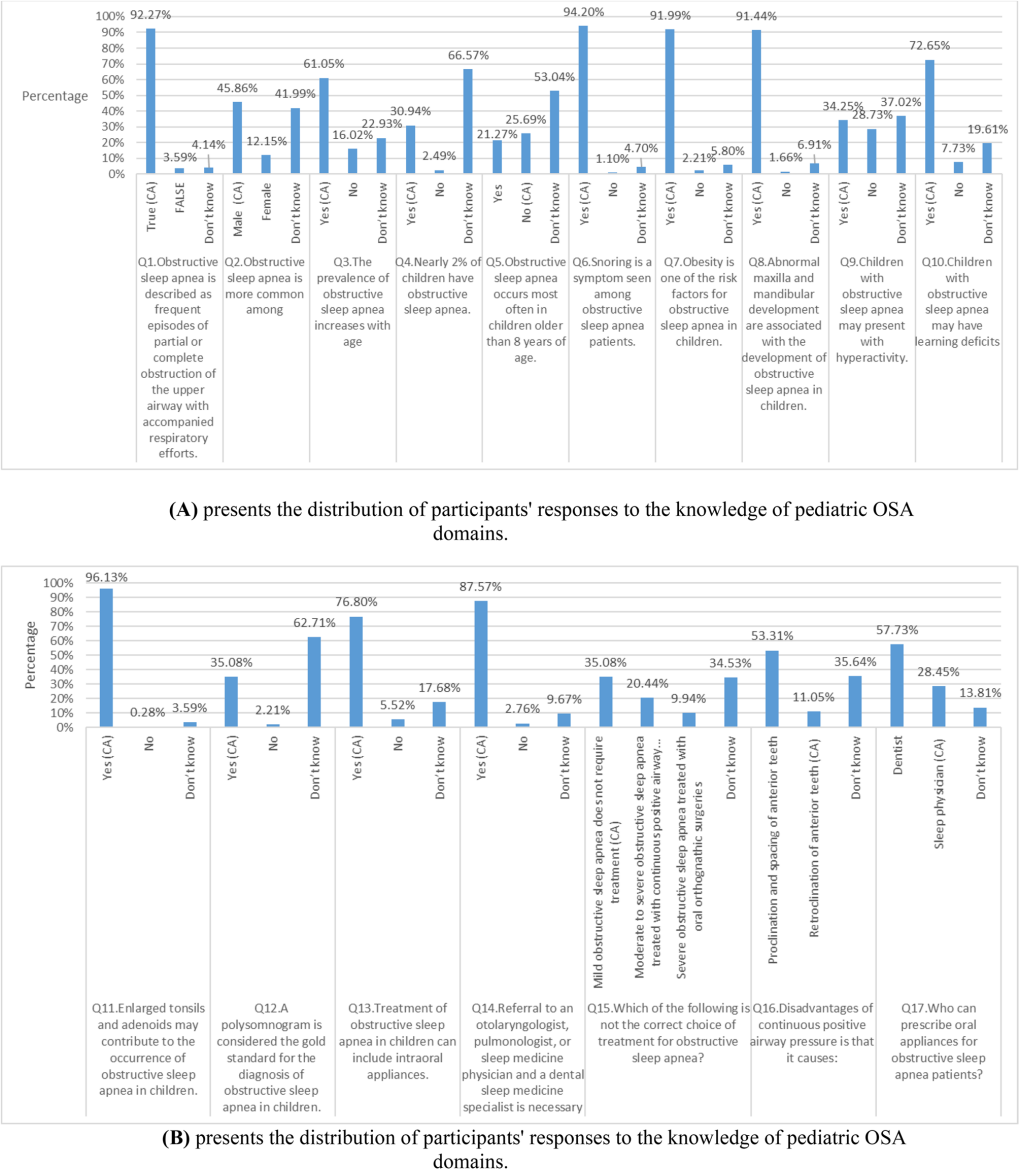
**A** Presents the distribution of participants' responses to the knowledge of pediatric OSA domains. **B** Presents the distribution of participants' responses to the knowledge of pediatric OSA domains

However, deficient knowledge was observed regarding gender predilection (Q. No.2), the prevalence of OSA (Q. No.4), the commonly affected age group (Q. No. 5), the general findings (Q. No. 9), and the diagnosis of OSA using polysomnography (Q. No. 12), treatment option for OSA (Q.15), disadvantage of continuous positive airway pressure (Q. No.16), and who can prescribe oral appliances (Q. No. 17) with the correct answer (CA) identified, as shown in Fig. 1A and B.

### Responses to the attitude section

**Fig. 2:**
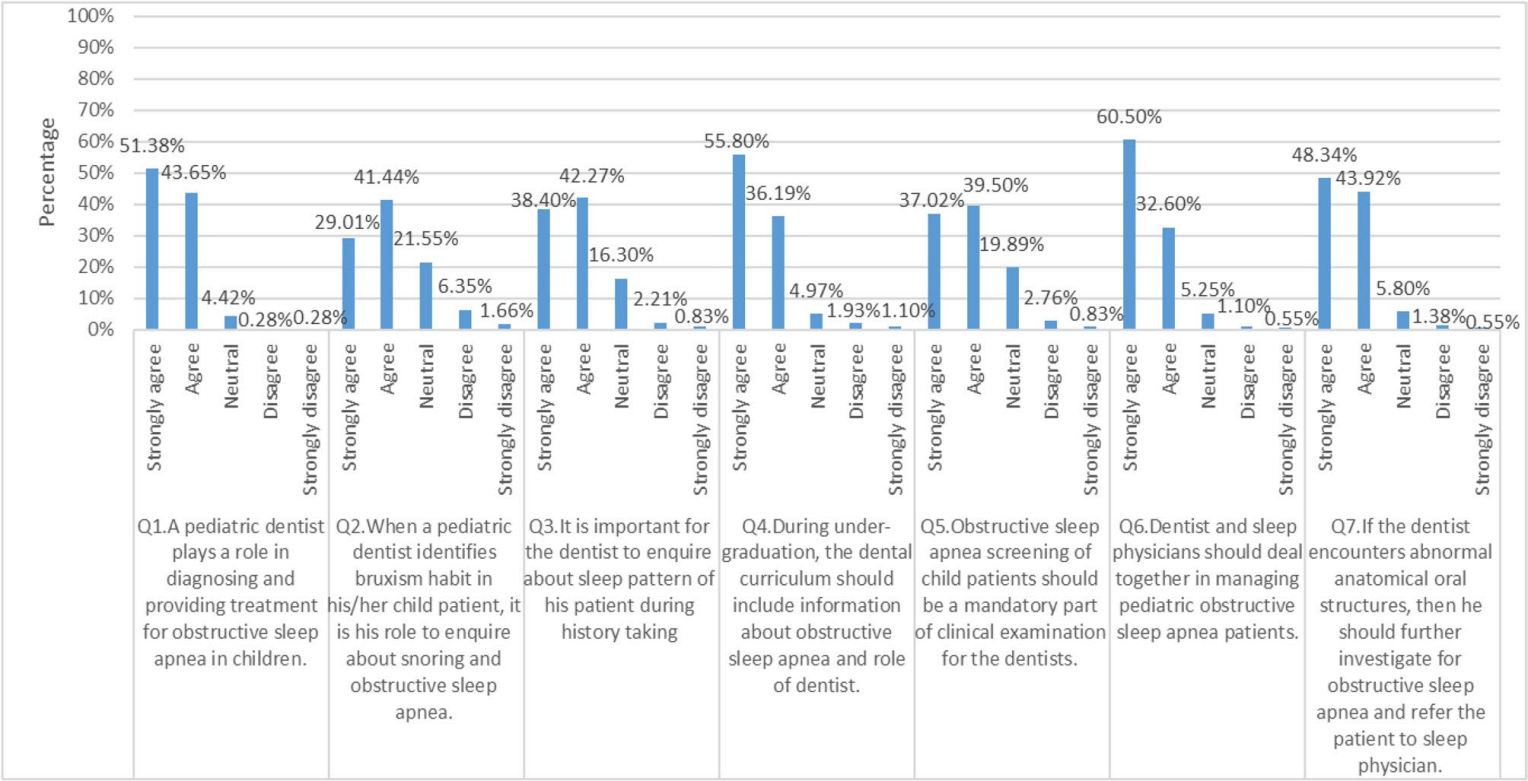
Presents the distribution of participants' responses to the attitude towards pediatric OSA domains

The present study demonstrated a positive attitude toward their involvement in OSA diagnosis and treatment (Q. No. 1, 2, 5), inquiring about patients’ sleep patterns (Q. No. 3), the integration of OSA-related knowledge into dental undergraduate education (Q. No.4), interdisciplinary approach working and referring cases with physicians (Q. No. 6, 7), as shown in Fig. 2.

### Responses to the practice section

**Fig. 3:**
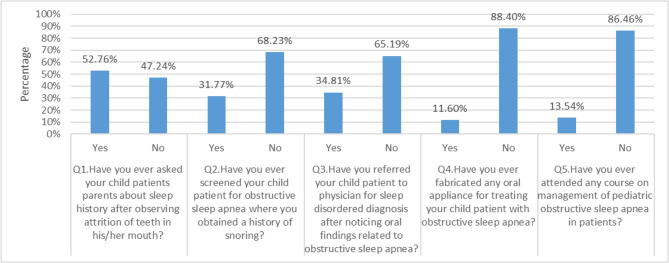
Presents the distribution of participants' responses to the practice of pediatric OSA domains

The participating dentists demonstrated adequate practice only regarding the inquiry about patients’ sleep history when observing attrition on teeth (Q. No. 1). However, practices across all other domains were lacking including screenings for history of snoring (Q. No. 2), referral for sleep-disordered diagnosis to a physician after noting oral findings linked to OSA (Q. No. 3), prior involvement in fabricating oral appliances for OSA cases (Q. No. 4) and previous attendance in OSA management sessions (Q. No. 5), as illustrated in Fig. 3.

### Overall knowledge, attitude, and practice of the participants

Overall, 310 (85.64%) pediatric dentists had adequate knowledge, and 343 (94.75%) demonstrated a positive attitude toward OSA. In contrast, poor practices were noted by 274 (75.69%) pediatric dentists, as described in Table 2.

**Table 2:** Distribution of knowledge, attitude, and practice categories among the study sample

Outcome	Category	Number	Percentages (%)
Knowledge	Adequate	310	85.64
	Inadequate	52	14.36
Attitude	Positive	343	94.75
	Negative	19	5.25
Practice	Good	88	24.31
	Poor	274	75.69

### Correlation between knowledge, attitude, and practice in relation to demographic variables

The correlation between knowledge, attitude, and practice in relation to demographic variables is presented in the Supplemental file (1). Regarding the correlation between knowledge, attitude, practice, and gender, no correlation was detected (Phi values= −0.044, 0.028, and 0.095, respectively), with no statistical significance (*p*-values = 0.407535, 0.588659, and 0.069874, respectively).

A negative correlation of weak strength (*r* = − 0.104) with statistical significance (*p*-value = 0.047186) was detected between practice and age. In comparison, a correlation of very weak strength (*r* = 0.02 and − 0.02, respectively) with no statistically significant difference was detected between knowledge, attitude, and age (*p*-values = 0.678455 and 0.759706, respectively).

Concerning the correlation between knowledge and years of experience, a weak correlation (*r* = 0.132) with a statistical significance (*p*-value = 0.011848) was detected. The correlation between attitude, practice, and years of experience showed weak strength (*r*= −0.10 and − 0.10, respectively) with no statistical significance (*p*-value = 0.050883 and 0.064170, respectively).

Results of the correlation between knowledge, attitude, practice, and practice sector showed a weak strength (λ = 0.007, 0.015, and 0.009, respectively) with no statistical significance (*p*-value = 0.486000, 0.148000, and 0.347000, respectively).

Finally, the correlation between knowledge, attitude, practice, and nationality showed a weak correlation (λ = 0.011, 0.020, and 0.021, respectively) with no statistical significance (*p*-value = 0.545000, 0.201000, and 0.189000, respectively).

## Discussion

The value of screening children for OSA cannot be adequately emphasized enough. It was estimated that 82–93% of adults with OSA are still undiagnosed. Similarly, OSA has lately been identified as one of the most frequent and underdiagnosed chronic childhood disorders. The distressing rise in youthful obesity over the previous few decades has been connected with an escalation in the prevalence of OSA from a steady 1–4% to as extreme as 19–61%, highlighting the urge for OSA screening in children by health care providers [17–19].

Pediatric dentists play a pivotal role in the early recognition, referring, advising, and treating patients with OSA. A patient’s routine dental visits and the dentist’s accessibility to examine the upper airway facilitate screening patients for OSA and identifying patients at greatest risk [20].

Insufficient knowledge of pediatric dentists regarding OSA may lead to expanded undiagnosed cases and related risks. Even though there is sufficient research on screening and treatment procedures, there is scarce research among pediatric dentists on the knowledge of OSA [13]. So, the present study aimed to evaluate pediatric dentists’ knowledge, attitude, and practice toward OSA among children in Egypt.

Our results showed that 85.64% of participants have adequate knowledge of OSA among children, which was in line with previous studies [15, 21, 22]. These findings can be attributed to the fact that pediatric dentists typically receive more comprehensive training in child development, medical conditions affecting children, and interdisciplinary care approaches [17]. Moreover, pediatric dentists are exposed to education on OSA through the integration of the American Academy of Pediatric Dentistry policy statement into their postgraduate studies, which showcases a considerable depth of knowledge in this domain [15].

In contrast, Alharbi et al. [7] and Alzahrani et al. [13] reported that the knowledge of participants is very limited, regardless of good identification of OSA as an essential clinical condition in children, and they attributed that to the dearth of experience of students and new graduates [7]. Besides, these findings accentuate the substantial diversity in knowledge levels across different populations, underscoring the paramount significance of educational initiatives and awareness campaigns to enhance the conception of OSA and its linked signs and symptoms within various fields of dentistry [15].

A closer view of the individual queries in this study showed that participants performed better in the questions about the definition, age predilection, signs and symptoms, risk factors of pediatric OSA, treatment with intra-oral appliances, and referral practices. These findings were in accordance with former studies [13, 23, 24], which can be justified by the more advanced educational level of the participants [13, 23].

Conversely, questions about epidemiology, gender predilection, common findings such as hyperactivity, diagnosis, and treatment were the most poorly performed, in agreement with Sawan et al. [24]. These findings can be linked to the absence of learning objectives for OSA in the dental curriculum, lack of clinical training, variability in postgraduate education, and delayed curricular integration of sleep medicine topics in dental education worldwide [14, 25].

Pediatric dentists displayed a constructive attitude towards screening and diagnosis, dental curriculum, and interdisciplinary approach, which agreed with [14, 26]. These findings can be linked to pediatric dentists’ recognized essential role in the early identification and management of pediatric OSA. They frequently encounter anatomical features and clinical signs that are critical indicators of pediatric OSA. This frontline position fosters a sense of responsibility and motivation to screen and diagnose OSA effectively. Moreover, there is an increased acknowledgment of the merits of interdisciplinary collaboration and the pursuit of pertinent medical expertise to facilitate the efficacious screening and management of OSA [15, 17, 27].

On the contrary, Alzahrani et al. [13] revealed a generally negative attitude of participants (63.35%) towards OSA. Most respondents thought screening patients for possible OSA is not essential because they were not confident in utilizing screening equipment, managing with oral appliances, and referring to the sleep medicine units [13].

The present study revealed dentists’ poor practices in screening, diagnosing, and treating OSA patients in their everyday dental practice and ongoing dental education. These findings agreed with Kale et al. [14] and Sawan et al. [24], which can be attributed to dentists considering OSA primarily as a medical condition separate from the domain of dentistry, and their desire to refer patients to specialized sleep physicians to avoid liability risk [13, 15]. Besides, the knowledge level of this subject among their patients may render them uncomfortable telling their snoring to a dentist [24].

Although the present study demonstrated adequate knowledge and good attitude among pediatric dentists toward OSA, their practice was poor, which may be attributed to several barriers faced by pediatric dentists in effectively managing OSA in children. These barriers include inadequate clinical training, limited access to appropriate diagnostic tools, and the lack of effective interdisciplinary coordination, which leads to fragmented care and postponed treatment [28–30].

Although polysomnography (PSG) remains the gold standard in diagnosing OSA, there are many concerns about its use, as the restricted number of sleep laboratories and the elevated cost of performing a PSG for every child who snores or who may be at risk [31, 32]. So, besides a thorough physical examination, the Pediatric Sleep Questionnaire (PSQ) can be successfully included in dental practice as an additional screening tool. Its use by pediatric dentists may allow early detection and prompt referral for thorough assessment and care of pediatric OSA cases [31, 33].

Concerning the correlation between the knowledge, attitude, and practice and demographic data, only a weak negative correlation with statistical significance was detected between practice and age, as well as a weak positive correlation was detected between knowledge and years of experience, with statistical significance, which was in accordance with Sawan et al. [24] who revealed an insignificant difference in the knowledge score based on gender and work sector. Similar findings were reported by Alzahrani et al. [13], who found no significant differences between the mean attitude and knowledge scores based on practice sector, professional title, or sex. These results can be attributed to factors other than demographics, such as the amount and quality of education and training received about OSA, which play a more critical role in shaping dentists’ knowledge, attitudes, and practice. Also, it suggests that the participants with good knowledge gained knowledge via self-study [7].

According to the findings of this study, it is essential to recommend the introduction of OSA at the undergraduate level to increase future dentists’ knowledge and proficiency. Pediatric dental professionals in practice should also take part in OSA-focused continuing education courses. Through these initiatives, pediatric dentists can significantly reduce the severe health effects of pediatric OSA.

### Study limitations

The current results cannot be generalized to other countries where dental education about the subject could differ significantly. An additional limitation would be that this was a cross-sectional study, which hinders causation from any observed associations. Still, the present research affords baseline data that guarantee further investigation in future studies.

## Conclusions

Although adequate knowledge and a positive attitude toward different domains of pediatric OSA seem to exist among pediatric dentists in Egypt, they still lack adequacy in practice. Good knowledge was favoured among experienced dentists, while poor practice was linked to younger dentists. Pediatric dentists may not properly diagnose children with OSA if they are not well-versed and proficient in screening procedures, treatment options, and the referral system. As a result, many cases can go undetected.

## Supplementary Information

Supplementary Material 1.

## Data Availability

All data generated or analyzed during the study are included in this research article.

## Abbreviations

OSA: Obstructive sleep apnea
PSQ: Pediatric Sleep Questionnaire
PSG: Polysomnography

## References

[1] Rojekar N, Sajjanar A, Kulkarni S, Bhattad D, Shukla H, Bhagwani D. Knowledge, attitude and practice of pedodontists, pediatricians and ENT surgeons towards pediatric obstructive sleep apnea: A cross sectional study. IJMHS. 2020;10(04):946–51. 10.15520/ijmhs.v10i03.282.

[2] Iannella G, Pace A, Bellizzi MG, Magliulo G, Greco A, De Virgilio A, et al. The global burden of obstructive sleep apnea. Diagnostics. 2025;15(9):1088. 10.3390/DIAGNOSTICS15091088. 40361906 10.3390/diagnostics15091088 PMC12071658

[3] Magnusdottir S, Hill EA. Prevalence of obstructive sleep apnea (OSA) among preschool aged children in the general population: a systematic review. Sleep Med Rev. 2024;73:101871. 10.1016/j.smrv.2023.101871. 37976758 10.1016/j.smrv.2023.101871

[4] Sayed-Ahmed MM, Taher MB, Zaytoun RA, Abdel Hady AF. Evaluation of sleep difficulties in Egyptian children with down syndrome: a case–control study. Indian J Otolaryngol Head Neck Surg. 2024;76(1):97–102. 10.1007/S12070-023-04090-9. 38440482 10.1007/s12070-023-04090-9 PMC10908933

[5] Ouda-Abdelateif A, Amin Mohammed E, Nader Sadek B. Sleep disorders among children suffering from bronchial asthma. Egypt J Health Care. 2022;13(3):324–35. 10.21608/EJHC.2022.251537.

[6] Arcidiacono L, Santagostini A, Tagliaferri S, Ghezzi B, Manfredi E, Segù M. Awareness and attitude among general dentists and orthodontists toward obstructive sleep apnea in children. Front Neurol. 2024;15:1279362. 10.3389/FNEUR.2024.1279362. 38445265 10.3389/fneur.2024.1279362 PMC10913199

[7] Alharbi LN, Alsaikhan MA, Al-Haj Ali SN, Farah RF. Pediatric obstructive sleep apnea: knowledge and attitudes of medical and dental students and fresh graduates from Saudi Arabia. Children. 2021;8(9):768. 10.3390/children8090768. 34572200 10.3390/children8090768 PMC8471539

[8] Kim JS, Choi JH. Pediatric obstructive sleep apnea: clinical manifestations and consequences. Korean J Otorhinolaryngol -Head Neck Surg. 2024;67(10):515–24. 10.3342/kjorl-hns.2024.00374.

[9] Shekarian M, Feizbakhsh M, Rafie M. Knowledge and attitude of general dentists, senior dental students, and orthodontic residents toward obstructive sleep apnea. Clin Exp Dent Res. 2024;10(5):e931. 10.1002/CRE2.931. 39295297 10.1002/cre2.931 PMC11411143

[10] Khan O, Zahid A, Mumtaz A, Shujat A. Sleep disorder: an obstructive sleep apnea, dentist Understanding about it. JHRR. 2023;3(2):236–40.

[11] Alkharouby SA, Alkhudhayri SL, Alhassani SL, Alghamdi HS, Alsahafi RA, Mariappan N, et al. General dentists and dental specialists’ knowledge of treatment, diagnosis, referral, and risk factors of obstructive sleep apnea: a systematic review. Dent J. 2025;13(5):187. 10.3390/DJ13050187. 10.3390/dj13050187 PMC12110612 40422607

[12] Pan Z, Ma T, Zeng Q, Xu T, Ran Q, Li T, et al. People’s knowledge, attitudes, practice, and healthcare education demand regarding OSA: a cross-sectional study among Chinese general populations. Front Public Health. 2023;11:1128334. 10.3389/FPUBH.2023.1128334. 37521967 10.3389/fpubh.2023.1128334 PMC10372425

[13] Alzahrani MM, Alghamdi AA, Alghamdi SA, Alotaibi RK. Knowledge and attitude of dentists towards obstructive sleep apnea. Int Dent J. 2022;72:315–21. 10.1016/J.IDENTJ.2021.05.004. 34193341 10.1016/j.identj.2021.05.004 PMC9275360

[14] Kale SS, Kakodkar P, Shetiya SH. Obstructive sleep apnea domains: knowledge, attitude and practice results of dentists from a dental college in India. Sleep Sci. 2020;13:3–9. 10.5935/1984-0063.20190121. 32670486 10.5935/1984-0063.20190121 PMC7347360

[15] Nair LR, George S, Anandaraj S, Peter J, Soorya R, Salim S. Knowledge, attitude, and practice regarding different domains of pediatric obstructive sleep apnea among pediatric dentists from kerala: A cross-sectional study. J Indian Soc Pedod Prev Dent. 2023;41(3):181–9. 10.4103/JISPPD.JISPPD_226_23. 37861631 10.4103/jisppd.jisppd_226_23

[16] Chauhan M, Chauhan MR, Agrawal A, Chawla AR. Gauging the knowledge, attitude and practice of interns and dentists about obstructive sleep apnoea—a crosssectional study. Lung India. 2024;41(1):25–9. 10.4103/LUNGINDIA.LUNGINDIA_201_23. 38160455 10.4103/lungindia.lungindia_201_23 PMC10883453

[17] Chiang HK, Reddy N, Carrico C, Best AM, Leszczyszyn DJ. The prevalence of pediatric dentists who screen for obstructive sleep apnea. J Dent Sleep Med. 2017;4(1):5–10. 10.15331/JDSM.6416.

[18] Adnan MM, Kassim NK, Rasid NMM, Ibrahim H, Ramli R, Ghani N, et al. Knowledge and attitude of obstructive sleep apnea among dental students in North East penisular Malaysia. Bangladesh J Med Sci. 2022;21(2):384–90. 10.3329/BJMS.V21I2.58071.

[19] Celejewska-Wójcik N, Polok K, Górka K, Stachura T, Kania A, Nastałek P, et al. Association between undiagnosed obstructive sleep apnea and severe course of COVID-19: a prospective observational study. Sleep Breath. 2024;28(1):79–86. 10.1007/S11325-023-02855-8. 37418221 10.1007/s11325-023-02855-8 PMC10954863

[20] American Academy of Pediatric Dentistry. Policy on Obstructive Sleep Apnea (OSA). The reference manual of pediatric dentistry: 2024–2025. Ill.: American Academy of Pediatric Dentistry;: Chicago; 2024. pp. 139–42.

[21] Fagundes NCF, da Rosa Moreira Bastos RT, Perez A, Flores-Mir C, Normando D. Practices and perception of paediatric obstructive sleep apnea among orthodontists and paediatric dentists in brazil: A Mixed-Methods study. Orthod Craniofac Res. 2024. 10.1111/ocr.12866. 39387694 10.1111/ocr.12866

[22] Díaz-Díaz MF, Schlaefli-Arrieta X, Caballero García S, Geller Palti D. Association between knowledge and attitudes towards pediatric obstructive sleep apnea and dental specialty. Cranio. 2025;43(4):678–87. 10.1080/08869634.2023.2281286. 37982430 10.1080/08869634.2023.2281286

[23] Vuorjoki-Ranta TR, Lobbezoo F, Vehkalahti M, Tuomilehto H, Ahlberg J. Treatment of obstructive sleep Apnoea patients in community dental care: knowledge and attitudes among general dental practitioners and specialist dentists. J Oral Rehabil. 2016;43(12):937–42. 10.1111/JOOR.12441. 27627187 10.1111/joor.12441

[24] Sawan N, Bakhsh H, Aldossary M, Alhussan R, Alharbi N, Abdellatif HM. Obstructive sleep apnea awareness among dentists in Saudi arabia: A Cross-Sectional study. Cureus. 2023;15(3). 10.7759/CUREUS.36463. 10.7759/cureus.36463 PMC10115731 37090274

[25] Herrero Babiloni A, Beetz G, Dal Fabbro C, Martel MO, Huynh N, Masse JF, et al. Dental sleep medicine: time to incorporate sleep apnoea education in the dental curriculum. Eur J Dent Educ. 2020;24(3):605–10. 10.1111/EJE.12533. 32320121 10.1111/eje.12533

[26] Jokubauskas L, Pileičikienė G, Žekonis G, Baltrušaitytė A. Lithuanian dentists’ knowledge, attitudes, and clinical practices regarding obstructive sleep apnea: a nationwide cross-sectional study. Cranio. 2019;37(4):238–45. 10.1080/08869634.2018.1437006. 29431599 10.1080/08869634.2018.1437006

[27] Giuca MR, Carli E, Lardani L, Pasini M, Miceli M, Fambrini E. Pediatric obstructive sleep apnea syndrome: emerging evidence and treatment approach. TSWJ. 2021;2021(1):5591251. 10.1155/2021/5591251. 10.1155/2021/5591251 PMC8088382 33981185

[28] Yu JL, Afolabi-Brown O. Updates on management of pediatric obstructive sleep apnea. Pediatr Investig. 2019;3(4):228–35. 10.1002/ped4.12164. 32851328 10.1002/ped4.12164 PMC7331384

[29] Mallappa JS, Arya R, Jojo A, Saisree AV, Vishal P, Pai KP, et al. Obstructive sleep apnea: A multidisciplinary challenge: the roles of pediatric dentists, orthodontists, and prosthodontists in diagnosis and management: A comprehensive review. Int J Appl Dent Sci. 2025;11(2):285–91. 10.22271/oral.2025.v11.i2d.2167.

[30] Holeyannavar RM, Kunder AS, Babaji P, KK S. Pediatric obstructive sleep apnea: A pedodontist’s perspective on causes, consequences and care. Int J Dent Sci. 2024;6(2):1–8.

[31] Chervin RD, Hedger K, Dillon JE, Pituch KJ. Pediatric sleep questionnaire (PSQ): validity and reliability of scales for sleep-disordered breathing, snoring, sleepiness, and behavioral problems. Sleep Med. 2000;1(1):21–32. 10.1016/S1389-9457(99)00009-X. 10733617 10.1016/s1389-9457(99)00009-x

[32] Mitchell M, Werkhaven JA. Cost-effectiveness of polysomnography in the management of pediatric obstructive sleep apnea. Int J Pediatr Otorhinolaryngol. 2020;133:109943. 10.1016/j.ijporl.2020.109943. 32086039 10.1016/j.ijporl.2020.109943

[33] Almutairi N, Alshareef W, Alhajress R, Almakoshi L, Zakzouk A, Aljasser A, et al. Translation and validation of the Arabic version of the sleep-related breathing disorder scale of the pediatric sleep questionnaire (PSQ-SRBD). Am J Otolaryngol. 2023;44(3):103805. 10.1016/J.AMJOTO.2023.103805. 36871419 10.1016/j.amjoto.2023.103805

