# Supplementary material for: Pediatric obstructive sleep apnea: knowledge, attitude, and practice among pediatric dentists in Egypt: a cross-sectional study
Source: BMC Oral Health. 2025 Sep 23;25:1417. doi: 10.1186/s12903-025-06944-w (PMC12459050; doi:10.1186/s12903-025-06944-w)
Supplement: Supplementary file 1 — Supplementary Material 1. [file 12903_2025_6944_MOESM1_ESM.docx]

**Supplemental file (1)**

*Correlation between* *knowledge, attitude, practice, and gender*

| Gender | Knowledge Level | | | Phi value | P-Value |
| --- | --- | --- | --- | --- | --- |
|  | Adequate | Inadequate | Total |  |  |
| Male | 130 (83.9%) | 25 (16.1%) | 155 (100%) | -0.044 | 0.407535 ns |
| Female | 180 (87.0%) | 27 (13.0%) | 207 (100%) |  |  |
| Total | 310 (85.6%) | 52 (14.4%) | 362 (100%) |  |  |
| Gender | **Attitude Level** | | | **Phi value** | **P-Value** |
|  | Positive | Negative | Total |  |  |
| Male | 148 (95.5%) | 7 (4.5%) | 155 (100%) | 0.028 | 0.588659 ns |
| Female | 195 (94.2%) | 12 (5.8%) | 207 (100%) |  |  |
| Total | 343 (94.8%) | 19 (5.2%) | 362 (100%) |  |  |
| Gender | **Practice Level** | | | **Phi value** | **P-Value** |
|  | Good | Poor | Total |  |  |
| Male | 45 (29.0%) | 110 (71.0%) | 155 (100%) | 0.095 | 0.069874 ns |
| Female | 43 (20.8%) | 164 (79.2%) | 207 (100%) |  |  |
| Total | 88 (24.3%) | 274 (75.7%) | 362 (100%) |  |  |

*Significant (s) at p ≤ 0.05, non-significant (ns) at p> 0.05.

*Correlation between knowledge, attitude, practice, and age*

| Age | Knowledge Level | | | Rank biserial correlation coefficient | P-Value |
| --- | --- | --- | --- | --- | --- |
|  | Adequate | Inadequate | Total |  |  |
| 25-34 Y | 202 (86.3%) | 32 (13.7%) | 234 (100.0%) | 0.02 | 0.678455 ns |
| 35-44 Y | 92 (83.6%) | 18 (16.4%) | 110 (100.0%) |  |  |
| 45-54 Y | 11 (91.7%) | 1 (8.3%) | 12 (100.0%) |  |  |
| More than 54 Y | 5 (83.3%) | 1 (16.7%) | 6 (100.0%) |  |  |
| Total | 310 (85.6%) | 52 (14.4%) | 362 (100.0%) |  |  |
| Age | **Attitude Level** | | | **Rank biserial correlation coefficient** | **P-Value** |
|  | Positive | Negative | Total |  |  |
| 25-34 Y | 221 (94.4%) | 13 (5.6%) | 234 (100.0%) | -0.02 | 0.759706 ns |
| 35-44 Y | 105 (95.5%) | 5 (4.5%) | 110 (100.0%) |  |  |
| 45-54 Y | 12 (100.0%) | 0 (0.0%) | 12 (100.0%) |  |  |
| More than 54 Y | 5 (83.3%) | 1 (16.7%) | 6 (100.0%) |  |  |
| Total | 343 (94.8%) | 19 (5.2%) | 362 (100.0%) |  |  |
| Age | **Practice Level** | | | **Rank biserial correlation coefficient** | **P-Value** |
|  | Good | Poor | Total |  |  |
| 25-34 Y | 50 (21.4%) | 184 (78.6%) | 234 (100.0%) | -.104* | 0.047186 s |
| 35-44 Y | 30 (27.3%) | 80 (72.7%) | 110 (100.0%) |  |  |
| 45-54 Y | 5 (41.7%) | 7 (58.3%) | 12 (100.0%) |  |  |
| More than 54 Y | 3 (50.0%) | 3 (50.0%) | 6 (100.0%) |  |  |
| Total | 88 (24.3%) | 274 (75.7%) | 362 (100.0%) |  |  |

*Significant (s) at p ≤ 0.05, non-significant (ns) at p> 0.05.

*Correlation between knowledge, attitude, practice, and* *years of experience*

| Years of experience | Knowledge Level | | | Rank biserial correlation coefficient | P-Value |
| --- | --- | --- | --- | --- | --- |
|  | Adequate | Inadequate | Total |  |  |
| Less than 5 Y | 85 (93.4%) | 6 (6.6%) | 91 (100.0%) | .132* | 0.011848 s |
| From 5 to 10 Y | 120 (85.1%) | 21 (14.9%) | 141 (100.0%) |  |  |
| More than 10 to 20 Y | 79 (80.6%) | 19 (19.4%) | 98 (100.0%) |  |  |
| More than 20 Y | 26 (81.3%) | 6 (18.8%) | 32 (100.0%) |  |  |
| Total | 310 (85.6%) | 52 (14.4%) | 362 (100.0%) |  |  |
| years of experience | **Attitude Level** | | | **Rank biserial correlation coefficient** | **P-Value** |
|  | Positive | Negative | Total |  |  |
| Less than 5 Y | 84 (92.3%) | 7 (7.7%) | 91 (100.0%) | -0.10 | 0.050883 ns |
| From 5 to 10 Y | 132 (93.6%) | 9 (6.4%) | 141 (100.0%) |  |  |
| More than 10 to 20 Y | 95 (96.9%) | 3 (3.1%) | 98 (100.0%) |  |  |
| More than 20 Y | 32 (100.0%) | 0 (0.0%) | 32 (100.0%) |  |  |
| Total | 343 (94.8%) | 19 (5.2%) | 362 (100.0%) |  |  |
| years of experience | **Practice Level** | | | **Rank biserial correlation coefficient** | **P-Value** |
|  | Good | Poor | Total |  |  |
| Less than 5 Y | 17 (18.7%) | 74 (81.3%) | 91 (100.0%) | -0.10  (Association of weak strength) | 0.064170 ns |
| From 5 to 10 Y | 33 (23.4%) | 108 (76.6%) | 141 (100.0%) |  |  |
| More than 10 to 20 Y | 28 (28.6%) | 70 (71.4%) | 98 (100.0%) |  |  |
| More than 20 Y | 10 (31.3%) | 22 (68.8%) | 32 (100.0%) |  |  |
| Total | 88 (24.3%) | 274 (75.7%) | 362 (100.0%) |  |  |

*Significant (s) at p ≤ 0.05, non-significant (ns) at p> 0.05.

*Correlation between* *knowledge, attitude, practice, and* *practice sector*

| Practice sector | Knowledge Level | | | Goodman and Kruskal's Lambda | P-Value |
| --- | --- | --- | --- | --- | --- |
|  | Adequate | Inadequate | Total |  |  |
| Academic work | 151 (86.3%) | 24 (13.7%) | 175 (100.0%) | 0.007 | 0.486000 ns |
| Primary health care hospital | 57 (86.4%) | 9 (13.6%) | 66 (100.0%) |  |  |
| Private | 92 (82.9%) | 19 (17.1%) | 111 (100.0%) |  |  |
| Others | 10 (100.0%) | 0 (0.0%) | 10 (100.0%) |  |  |
| Total | 310 (85.6%) | 52 (14.4%) | 362 (100.0%) |  |  |
| Practice sector | **Attitude Level** | | | **Goodman and Kruskal's Lambda** | **P-Value** |
|  | Positive | Negative | Total |  |  |
| Academic work | 163 (93.1%) | 12 (6.9%) | 175 (100.0%) | 0.015 | 0.148000 ns |
| Primary health care hospital | 66 (100.0%) | 0 (0.0%) | 66 (100.0%) |  |  |
| Private | 104 (93.7%) | 7 (6.3%) | 111 (100.0%) |  |  |
| Others | 10 (100.0%) | 0 0.0% | 10 (100.0%) |  |  |
| Total | 343 (94.8%) | 19 (5.2%) | 362 (100.0%) |  |  |
| Practice sector | **Practice Level** | | | **Goodman and Kruskal's Lambda** | **P-Value** |
|  | Good | Poor | Total |  |  |
| Academic work | 44 (25.1%) | 131 (74.9%) | 175 (100.0%) | 0.009 | 0.347000 ns |
| Primary health care hospital | 12 (18.2%) | 54 (81.8%) | 66 (100.0%) |  |  |
| Private | 31 (27.9%) | 80 (72.1%) | 111 (100.0%) |  |  |
| Others | 1 (10.0%) | 9 (90.0%) | 10 (100.0%) |  |  |
| Total | 88 (24.3%) | 274 (75.7%) | 362 (100.0%) |  |  |

*Significant (s) at p ≤ 0.05, non-significant (ns) at p> 0.05.

| Nationality | Knowledge Level | | | Goodman and Kruskal's Lambda | P-Value |
| --- | --- | --- | --- | --- | --- |
|  | Adequate | Inadequate | Total |  |  |
| Egyptian | 280 (85.4%) | 48 (14.6%) | 328 (100.0%) | 0.011 | 0.545000 ns |
| Palestinian | 6 (100.0%) | 0 (0.0%) | 6 (100.0%) |  |  |
| Saudi | 5 (100.0%) | 0 (0.0%) | 5 (100.0%) |  |  |
| Sudanese | 10 (76.9%) | 3 (23.1%) | 13 (100.0%) |  |  |
| Syrian | 6 (100.0%) | 0 (0.0%) | 6 (100.0%) |  |  |
| Yemeni | 3 (75.0%) | 1 (25.0%) | 4 (100.0%) |  |  |
| Total | 310 (85.6%) | 52 (14.4%) | 362 (100.0%) |  |  |
| Nationality | **Attitude Level** | | | **Goodman and Kruskal's Lambda** | **P-Value** |
|  | Positive | Negative | Total |  |  |
| Egyptian | 313 (95.4%) | 15 (4.6%) | 328 (100.0%) | 0.020 | 0.201000 ns |
| Palestinian | 5 (83.3%) | 1 (16.7%) | 6 (100.0%) |  |  |
| Saudi | 4 (80.0%) | 1 (20.0%) | 5 (100.0%) |  |  |
| Sudanese | 11 (84.6%) | 2 (15.4%) | 13 (100.0%) |  |  |
| Syrian | 6 (100.0%) | 0 (0.0%) | 6 (100.0%) |  |  |
| Yemeni | 4 (100.0%) | 0 (0.0%) | 4 (100.0%) |  |  |
| Total | 343 (94.8%) | 19 (5.2%) | 362 (100.0%) |  |  |
| Nationality | **Practice Level** | | | **Goodman and Kruskal's Lambda** | **P-Value** |
|  | Good | Poor | Total |  |  |
| Egyptian | 77 (23.5%) | 251 (76.5%) | 328 (100.0%) | 0.021 | 0.189000 ns |
| Palestinian | 2 (33.3%) | 4 (66.7%) | 6 (100.0%) |  |  |
| Saudi | 2 (40.0%) | 3 (60.0%) | 5 (100.0%) |  |  |
| Sudanese | 2 (15.4%) | 11 (84.6%) | 13 (100.0%) |  |  |
| Syrian | 2 (33.3%) | 4 (66.7%) | 6 (100.0%) |  |  |
| Yemeni | 3 (75.0%) | 1 (25.0%) | 4 (100.0%) |  |  |
| Total | 88 (24.3%) | 274 (75.7%) | 362 (100.0%) |  |  |

*Correlation between knowledge, attitude, practice, and* *Nationality*

*Significant (s) at p ≤ 0.05, non-significant (ns) at p> 0.05.
